# Identification of Candidate Gene for Internode Length in Rice to Enhance Resistance to Lodging Using QTL Analysis

**DOI:** 10.3390/plants10071369

**Published:** 2021-07-05

**Authors:** Dan-Dan Zhao, Ju-Hyeong Son, Muhammad Farooq, Kyung-Min Kim

**Affiliations:** Division of Plant Biosciences, School of Applied Biosciences, College of Agriculture and Life Science, Kyungpook National University, Daegu 41566, Korea; qx288mm@naver.com (D.-D.Z.); ff0319@hanmail.net (J.-H.S.); mfarooqsr@gmail.com (M.F.)

**Keywords:** doubled haploid, gibberellins, internode length, QTL, rice, stem diameter

## Abstract

Internode length and stem diameter are the primary traits affecting the lodging resistance of rice. Traits related to the length of the panicle (LP), uppermost internode (LUI), second internode (LSI), third internode (LTI), fourth internode (LFI), lowest internode (LLI) as well as stem diameter at the uppermost internode (SDUI), second internode (SDSI), third internode (SDTI), fourth internode (SDFI), and lowest internode (SDLI) in 120 Cheongcheong/Nagdong doubled haploid population were investigated using a quantitative trait locus (QTL) analysis. Thirty-four QTL regions affected LP and the length of each internode. Twenty-six QTL regions were associated with the stem diameter of each internode. RM12285-RM212 on chromosome 1 contained 10 QTLs related to the internode length, which have overlapped for over 2 years. Twenty-three candidate genes were screened using mark interval. Among the candidate genes, *Os01g0803900*, named *OsCYPq1*, which is in the *Cytochrome P450 family*, might be involved in gibberellins (GA) synthesis. GA is an essential plant growth regulator that affects plant height. *OsCYPq1* catalyzes oxidation steps in the middle part of the GA pathway. *OsCYPq1* is expected to provide valuable information to improve the marker assessment for target traits and QTL gene cloning in rice.

## 1. Introduction

Rice is a globally important food and a nutrition-rich crop. It feeds >50% of the world’s population. As the most important crop, its demand increases daily, increasing its attention in the scientific community to develop healthier and more productive rice cultivar to meet the feeding demand of the world. However, with the recent deterioration of the environment and the increasing number of typhoons, rice lodging has become serious [1]. Among these environmental conditions, lodging is considered one of the most affected environmental conditions that makes harvesting difficult and causes great loss of grain yield and quality [2]. Lodging primarily occurs at the stage when the plant’s stem is too weak to support the weight of its grain, and during that time, a small fraction of wind can also cause lodging [3]. Previous studies have shown that stem diameter and plant height are selectively correlative to the lodging resistance in rice [4,5,6]. During the Green Revolution, high-yielding varieties of rice and wheat were developed at suitable height levels [7,8,9], but not enough to cope with the current environmental degradation. To solve this problem, we need more robust rice varieties. Shorter plants spend fewer resources on stem growth, which can help increase stem diameter, thereby improving lodging resistance and yield [10]. The plant height of rice is determined by internode length, which has an important effect on the height of the center of gravity and the lodging resistance of the stem. The elongation in the internode is thought to be caused by two regulatory mechanisms [11]. The first one is the increase in cell division in the meristem region, which is regulated by GA-induced expression of cyclin genes and p34cdc2-like histone H1 kinase [12], and the second one is regulation by microtubule orientation; creep of cell wall polymers; and biosynthesis, transport, and consolidation of new cell wall components [13]. Therefore, understanding the molecular mechanism and to clear the signal network of regulating plant height is particularly important. Quantitative trait locus (QTL) mapping is the primary way to screen and clone trait-related genes in rice [14]. QTL analysis of plant height has recently been reported continuously. Ishimaru et al. [15] reported that total plant length was mapped to chromosome 1 in a backcross inbred line of Nipponbare and Kasalath. The QTL region of this population overlaps with the QTL of *semidwarf-1*. *Gibberellin 20-oxidase 2* (*Os01g0883800*) is one of the most famous genes affecting the height of rice, located on chromosome 1. This mutation resulted in the loss of function of the *semidwarf-1* QTL, resulting in reduced GA1 and shortening of the plant [16]. However, several plant height genes have been identified in the QTL region on chromosome 1. Tanaka et al. reported the gene responsible for three brittle mutants of rice by insertion of *Tos17*, in response to CesA (cellulose synthase catalytic subunit). Therefore, the isolation and characterization of the mutation inhibiting *Os01g0750300*, which is cellulose synthase catalytic subunit 4 gene, reduced the plant height [17]. Another known gene about height is *Os01g0853400* (Coronatine-insensitive protein 1), which can increase the elongated internodes by knockdown of expression [18].

Therefore, this study used a doubled haploid population from a cross between an *Indica* variety “Cheongcheong” and a *japonica* variety “Nagdong”, and further analyzed the mechanism affecting plant height according to agronomic traits, such as the length of each internode (LP, LUI, LSI, LTI, LFI, and LLI) and the stem diameter of each internode (SDUI, SDSI, SDTI, SDFI, and SDLI), using QTL analysis. In addition, at the QTL level, this study specifies the genetic relationship between the length of each internode and the stem diameter of each internode. Through QTL analysis, candidate genes related to internode length and stem diameter were screened. Among them, *OsCYPq1*, which is a cytochrome P450 gene, was selected as the candidate gene.

## 2. Results

### 2.1. Comparison of Agricultural Characteristics and Phenotypic Evaluation

**Figure 1:**
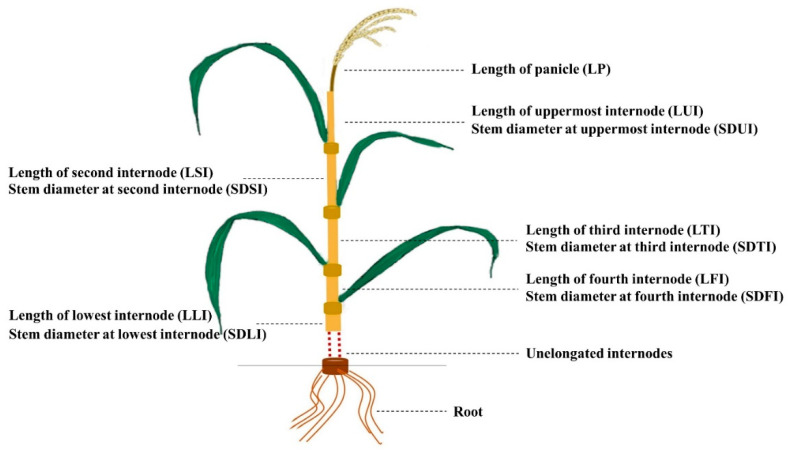
The image shows the location of each sampled part of the rice plant.

**Figure 2:**
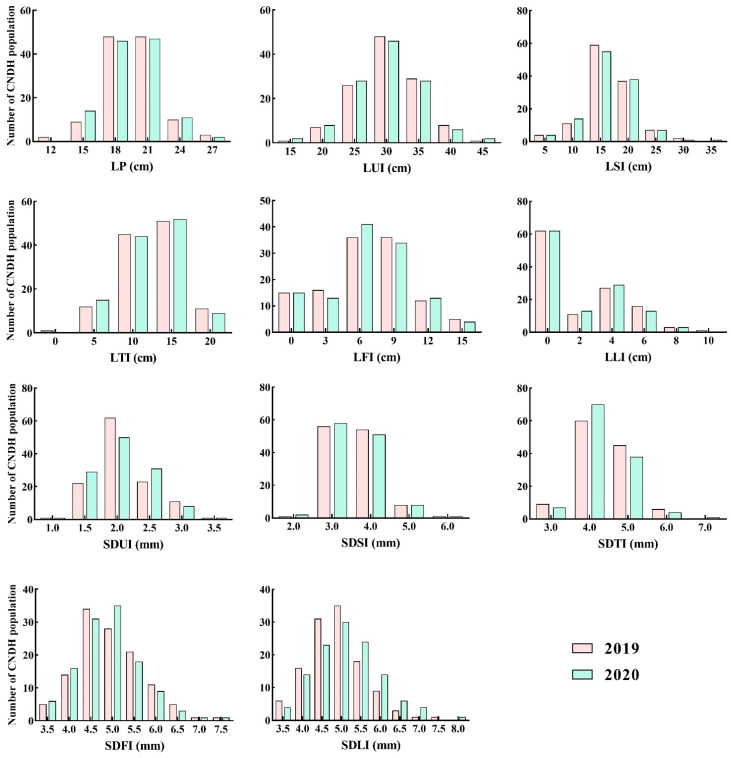
Frequency distribution of the length of each internode and the stem diameter of each internode in the Cheongcheong/Nagdong doubled haploid (CNDH) population. LP, length of the panicle; LUI, length of the uppermost internode; LSI, length of the second internode; LTI, length of the third internode; LFI, length of the fourth internode; LLI, length of the lowest internode; SDUI, stem diameter at the uppermost internode; SDSI, stem diameter at the second internode; SDTI, stem diameter at the third internode; SDFI, stem diameter at the fourth internode; SDLI, stem diameter at the lowest internode.

The plant phenotypes, such as the length of each internode and stem diameter of each internode (Figure 1) in 120 Cheongcheong/Nagdong doubled haploid (CNDH) population, Cheongcheong, and Nagdong were observed. Based on the two years of data, comparative analysis of the length of each internode and the stem diameter of each internode, it was observed that Cheongcheong were higher than Nagdong for all investigated traits (Table 1). Moreover, the curves obtained for the frequency distribution of the CNDH population were more likely to be similar to that of the normal distribution (Figure 2). It was confirmed that all examined agricultural characteristics were quantitative traits that one or more genes may control. In 2019 and 2020 analysis of correlation, each internode length has a high correlation with the stem diameter of each internode; however, for LUI, LFI, and LLI, there was no significant correlation with SDUI (Table S1).

### 2.2. QTL Analysis Associated with the Length and Stem Diameter of Each Internode

**Figure 3:**
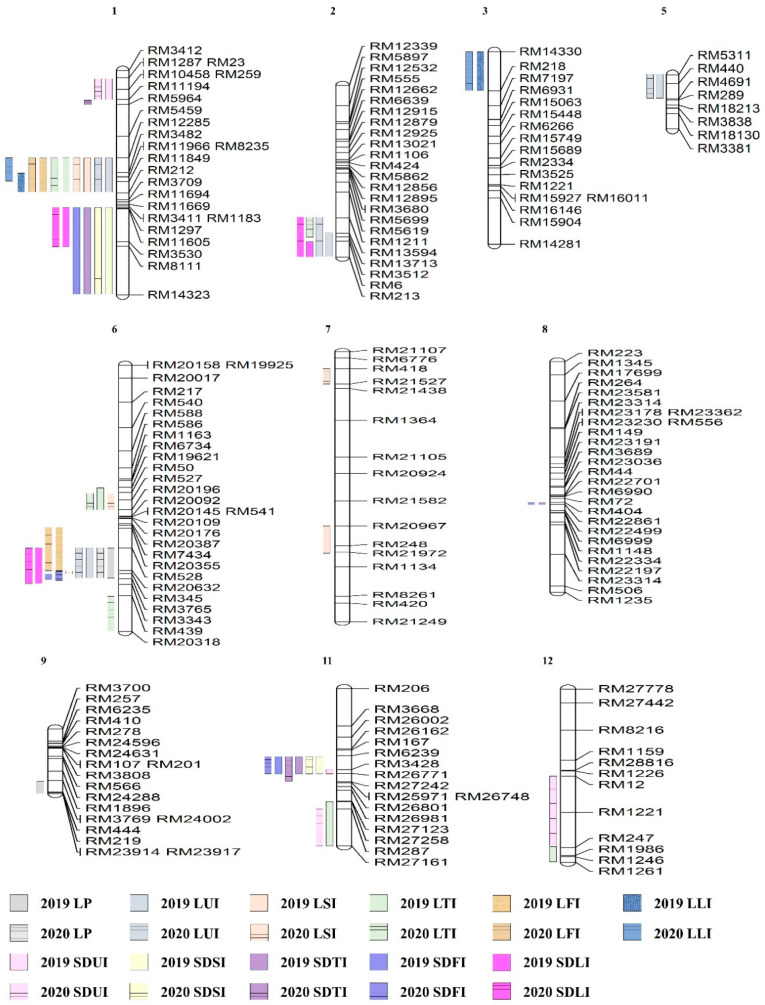
The chromosomal location of QTL is associated with each internode’s length and the stem diameter of each internode in the Cheongcheong/Nagdong doubled haploid (CNDH) population.

The phenotypic evaluation was recorded for four plants of each line. The average length and stem diameter of each internode was taken to be used for QTL analysis. In both 2019 and 2020, LP detected on chromosomes 6 and 9 possessed the overlap marker interval on RM528-RM3765, a higher LOD score of 3.64, and allele derived from Cheongcheong with 28% phenotypic variation. LUI, LSI, LTI, LFI, and LLI detected 34 QTLs on chromosomes 1, 2, 3, 5, 6, 7, 11, and 12. Significant QTL marker intervals were detected on chromosome 1 RM12285-RM212 for LUI, LSI, LTI, LFI, and LLI in the past two years. Among them, the phenotypic variation was 57% and showed positive effects from the Cheongcheong allele with the highest LOD 15.17. For SDUI, SDSI, SDTI, SDFI, and SDLI, 26 QTLs were detected on chromosomes 1, 2, 6, 8, 11, and 12. On the marker of interval RM6239-RM26771 on chromosome 6, there were regions related to SDSI, SDTI, and SDFI that overlapped in 2019 and 2020 (Figure 3, Table S2).

### 2.3. Based on QTL Mapping Search Candidate Gene

**Figure 4:**
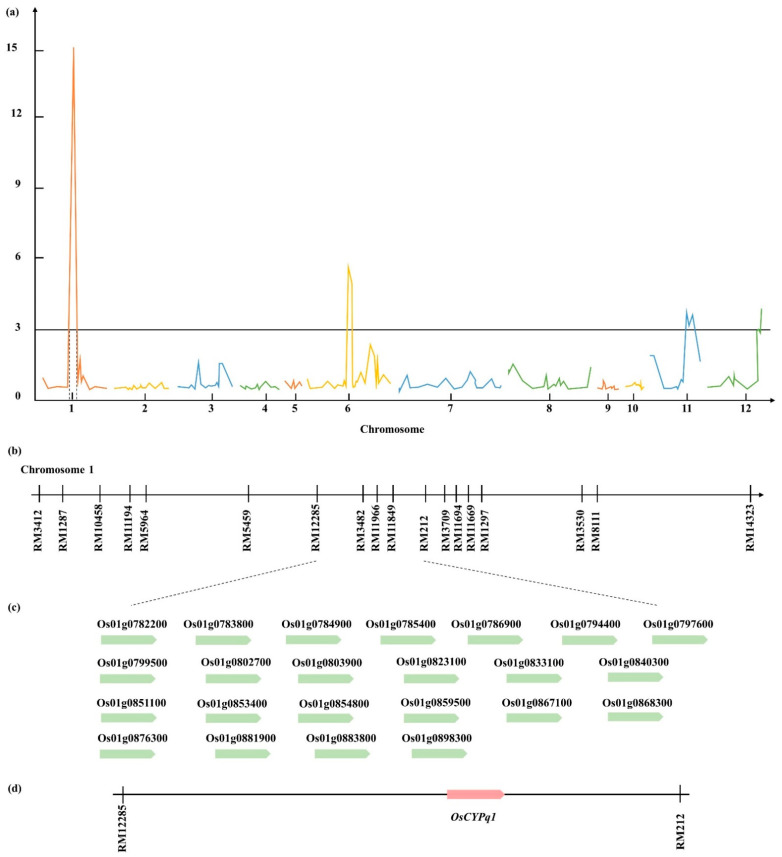
Quantitative trait locus (QTL) analysis and physical mapping of the gene related to the length of the internode. (**a**) Representative QTL analysis for LTI with the highest LOD value 15.17 on chromosome 1 in 2019 was selected. (**b**) The target marker interval RM12285-RM212 on chromosome 1. (**c**) Twenty-three candidate genes were found on chromosome 1 RM12285-RM212. (**d**) Among them, *OsCYPq1* was screened as a target gene related to the length of the internode.

QTL analysis for LUI, LSI, LTI, LFI, and LLI detected 10 QTLs on chromosome 1, between RM12285 and RM212, over the two years. NCBI screened the marker interval RM12285-RM212 of all ORFs. Twenty-three ORFs were associated with the length of the internode (Table 2). Their gene function classified all ORFs. Eleven candidate genes were involved in hormone signaling, and 12 candidate genes were related to cell function. The candidate genes involved in hormone signaling consisted of genes *cytochrome P450 family protein (CYP450)*, *auxin efflux carrier family protein*, *gibberellin 20 oxidase 2*, *GH3 auxin-responsive promoter family protein*, *leucine-rich repeat cysteine-containing* *protein*, *Axi 1-like protein*, *thioredoxin domain 2 containing protein*, *WD40-like domain protein*, *Cytochrome P450 86A1*, *similar to coronatine-insensitive protein 1*, and *similar to basic leucine zipper protein*. The candidate genes related to cell function include genes *alpha-expansin OsEXPA2*, *aminoacyl-tRNA synthetase Ib class domain protein*, *armadillo-like helical domain-containing protein*, *bHLH (basic helix-loop-helix) dimerization region domain protein*, *curculin-like (mannose-binding) lectin domain protein*, *diacylglycerol kinase catalytic region protein*, *DNA glycosylase family protein*, *ethylene-responsive element binding factor3 (OsERF3)*, *galactose oxidase*, *central protein*, *homeobox protein*, *NLI interacting factor protein*, and *DNA polymerase alpha catalytic subunit*. Among the candidate genes, the *Cytochrome P450 family* (*Os01g0803900*), which is involved in GA synthesis, was selected as the target gene (Figure 4).

### 2.4. The Homology Sequence of Candidate Gene and Phylogenetic Tree Analysis

**Figure 5:**
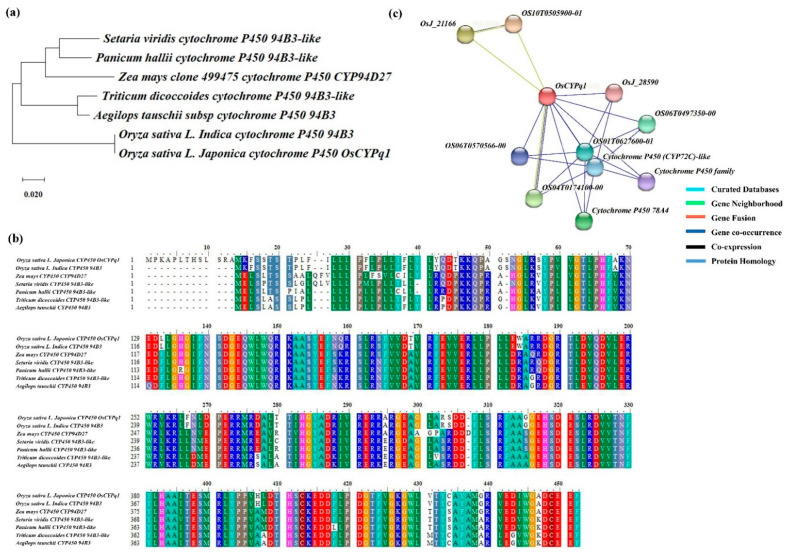
Sequence analysis of *OsCYPq1*. (**a**) The gene and homology genes were analyzed by a phylogenetic tree. The parsimony method with 1000 bootstrap replicates was used for constructing the phylogenetic tree. (**b**) Comparing the multiple sequence alignment of *OsCYPq1*, high similarity was found among *Oryza sativa* L., *Aegilops tauschii*, *Triticum dicoccoides*, *Zea mays*, *Panicum hallii*, and *Setaria viridis*. (**c**) *OsCYPq1* interacts with Cytochrome P450 family proteins. These are all involved in the GA synthesis pathway.

The target candidate gene, *Cytochrome P450*, also named as *OsCYPq1*, detected RM12285-RM212 on chromosome 1. Furthermore, the BLAST analysis by NCBI showed that *OsCYPq1* has a similar sequence with the *CYP450 94B3 of Oryza stative L. Indica*, *CYP450 CYP94D27* of *Zea mays*, *CYP450 94B3* of *Setaria viridis*, *Panicum hallii*, *Triticum dicoccoides*, and *Aegilops tauschii*. The genetic similarity was confirmed by phylogenetic tree analysis between the *OsCYPq1* and *Oryza stative L. Indica*, *Zea mays*, *Setaria viridis*, *Panicum hallii*, *Triticum dicoccoides*, *Aegilops tauschii* have a high genetic similarity. Moreover, using the domain of *OsCYPq1* to predict the functional partners, the result portrayed the interaction between *OsCYPq1* and 10 different proteins (Figure 5).

## 3. Discussion

Plant height is an essential characteristic of rice. Dwarf rice usually shows higher resistance to lodging and better soil nutrient usage, which can increase yield. Therefore, proper plant height is a prerequisite for optimal yield in rice breeding programs. It is necessary to elucidate the genetic basis of plant height to improve rice yield. Biologically, the height of a rice plant is equal to the length of the panicle plus its internode length from the ground. Combining these components makes it possible to determine the type of plant needed partially. Shearman et al. identified height QTLs on chromosome 1 using Chromosome Segment Substitution Line, and the significant QTL peaks were observed at 38.4 Mb [10]. This result is consistent with the current study, and many genes related to plant height were identified in this region. Therefore, we propose that chromosome 1 still has many genes controlling internode length and the underlying cause remains unknown. A reduction in plant height generally allows the plant to spend fewer resources on stem growth and helps increase the stem diameter, thereby improving lodging resistance and yield. Therefore, in this study, QTL analysis was performed by observing the length of each internode and the stem diameter of each internode. Based on correlation analysis, a very strong correlation between the length of the internode and the stem diameter was observed. According to the QTL analysis results, no QTL with internode length and stem diameter overlapping was found on chromosome 1. However, for the stem diameter of each internode, six significant QTLs were detected on chromosome 1 RM1297-RM8111. These results suggest that chromosome 1 is the key to control internode length and stem diameter in rice. Similarly, previous studies on internode length using Koshihikari and Kasalath backcrossing BC_1_F_3_ population detected 17 QTLs by QTL analysis, among QTLs most overlapped on chromosomes 1, 6, and 12 [19]. Moreover, Hattori et al., 2008 researched deepwater rice internode elongation by QTL analysis and detected the QTLs on chromosomes 1, 3, and 12 [20]. Therefore, the candidate genes were screened on chromosome 1 RM12285-RM212 with highly significant QTL peak, cytochrome P450 (*OsCYPq1*) gene, which is involved in the GA biosynthesis pathway, selected as a target gene.

The plant hormone GA is considered the most important class of plant growth regulators. It is the most important plant hormone for determining plant height [21]. It is synthesized through a complex biosynthesis pathway, which involves three classes of enzymes in a different cellular component [22]. One of these enzymes is the cytochrome P450s, the third-largest gene family and enzyme involved in plant metabolism, growth and development, and abiotic stress resistance, and represents 1% of the total plant’s protein coded genes [22,23].

It is well known that cytochrome P450 protein plays a vital role in a number of developmental processes through the biosynthesis or catabolism of phytohormones and other secondary compounds. For example, *CYP51G* can catalyze the essential 14α-demethylation of obtusifoliol, which is required for the synthesis of phytosterol and membrane sterols [24]. Similarly, *CYP85* a member of the cytochrome P450 performs functions in the modification of sterols and cyclic terpenes in brassinosteroid (BR), abscisic acid, and GA pathways [25,26]. Furthermore, the cytochrome P450 is mostly involved in BR synthesis and metabolism. The BR synthesis pathway has developed through the metabolism and analysis studies of dwarf mutants of the BR-deficient, shown to be defective in P450 genes, which indicates that P450 enzymes are involved in many steps in BR biosynthesis downstream of campesterol [27] and that those BRs are an important factor involved in plant growth and development.

In addition, it was estimated that there are 334 P450 genes in rice, 245 in Arabidopsis, 337 in soybean, and 270 in tomato plants [28]. The function of these cytochrome P450 families is unknown in many plant species. Many mutants of cytochrome P450-mediate steps in various plant spices have been crucial for isolating these genes encoding enzymes, which play a vital role in understanding the GA physiology [29]. Similarly, Zhu et al. found that recessive tall rice mutant with elongated upper internode are gene-encoded as monooxygenase *CYP714D1* have the capacity to catalyze the 16a,17-epoxidation non-13-hydroxylated GAs such as GA4, GA12, and GA9 to reduce the activity of GA biosynthesis in rice [30]. Large amounts of bioactive GAs are involved in the uppermost internode, encoding a P450 that deactivates GAs via GA 16a,17-epoxidation. GAs deactivation reaction has been GA 2-oxidation and catalyzed by soluble 2-oxoglutarate-dependent dioxygenases. Some 2b-hydroxylated GAs, including GA8, GA29, GA34, and GA51, were detectable in the uppermost internode. Thus, 2-oxidation of GA takes place in the uppermost internode tissue. However, the GA1 and GA4 in the elongated uppermost internode mutants illustrate that GA 2-oxidase activity is clearly insufficient to deactivate a fraction of GA pools that are normally metabolized by EUI in wild-type plants. These observations suggest that the *CYP450* gene family may have shown a positive role in the internode elongation and this can be a more useful tool in breeding technology to improve the internode formation of plants [30].

As cytochrome P450 is involved in the GA biosynthesis pathway, it may be a positive aspect to generate a semi-dwarf rice cultivar with a more productive and higher stem diameter [31]. Moreover, in rice, the elongation of the internode is considered one of the most essential traits to determine the plant height and grain yield. The internode elongation is under genetic control with various factor implications in the process [32]. Likewise, it was reported in rice that the *EUI1* is a key regulator of the elongation of the uppermost internode that encodes a cytochrome P450 protein (*CYP714D1*) and its expression level is related to GA-mediated SLR1 destruction. Furthermore, the feedback regulation of GA biosynthesis and *EUI1* plays a negative role in GA-mediated regulation of the elongation of rice upper internode cells [32]. In addition, Wang et al. identified a *CYP85A1* gene that belongs to the Cytochrome P450 family, which encodes BR-C6-oxidase in the BR biosynthesis pathway, and concluded that *CYP85A1* might have a positive role in internode elongation in cucumber plants [33]. Likewise, the result of polygenetic tree analysis shows that *OsCYPq1* is identical to the other plant’s cytochrome P450 family genes. The sequence analysis of *OsCYPq1* was found have a high similarity with Zea mays. Similarly, in 1995, Winkler and Helentjaris researched the maize *Dwarf3* gene that encodes a cytochrome P450 involved in an early step in GA biosynthesis [34]. Moreover, Magome et al. reported two cytochrome P450 family genes, *CYP714B1* and *CYP714B2*, encoded as GA 13-oxidase that reduced GA activity in rice plants. Their results revealed that *CYP714B1* and *CYP714B2* proteins can convert the GA_12_ to GA_53_. These results fully demonstrate the importance of the cytochrome P450 in GA biosynthesis.

Furthermore, *CYP714B1* and *CYP714B2* play an important role in GA13-hydroxylation found in rice and show that these mutants exhibit a normal appearance until the heading date, but show elongation in the uppermost internode upon the heading date, from which it was concluded that *CYP714B1* and *CYP714B2* play a vital role in GA 13-oxidase and plant growth and development as well as causing a decrease in GA activity. Ayano et al. reported that GA biosynthesis is important for internode elongation in deepwater rice [35]. However, all the above statements and our recent data obtained from QTLs analysis revealed that the *OsCYPq1* gene may have been playing a positive role in the rice plant internode formation, stem elongation, and an important role in the deactivation of bioactive Gas. It is a well-known phenomenon that GAs play an important role in the plant’s growth and development, which can be associated with the cytochrome P450 family that is involved in GA biosynthesis. Mutant plants with cytochrome P450 enzyme may play vital roles in rice plants by generating semi-dwarf rice that can overcome lodging resistance with more significant yield and productivity.

## 4. Materials and Methods

### 4.1. Plant Material and Field Experiment Design

A set of 120 CNDH populations were developed by doubled haploid from a cross between Cheongcheong (*O. sativa* L. ssp. *Indica*) and Nagdong (*O. sativa* L. ssp. *Japonica*). The genetic map was constructed by anther culture using an F_1_ population derived from the cross between Cheongcheong and Nagdong [36]. The experiment was conducted for 2 years in the experimental field (Hyoryeong-myeon, Gunwi-gun, Gyeongbuk, Korea. 36°11′ N, 128°64′ E). Before sowing, the seeds were surface-sterilized with 25% prochloraz (Hankook Samgong, Seoul, Korea) and soaked in tap water at 33 °C for 3 days in an incubator under dark conditions. The plants were transplanted on 24 May 2019, and 24 May 2020, in a randomized block design after 30 days of sowing. The 120 CNDH population and their parents were transplanted at 30 × 15 cm plant distance. All field management followed regular agricultural practices. To control pests and diseases, insecticides and herbicides were used following standard cultivation methods for Rural Development Administration. The amounts of the N, P_2_O_5_, and K_2_O fertilizers used were 9, 4.5, and 5.7 kg per 10 ha, respectively.

### 4.2. Phenotype Evaluation

The major agricultural traits associated with plant height (LP, LUI, LSI, LTI, LFI, and LLI) and the stem diameter of each internode (SDUI, SDSI, SDTI, SDFI, and SDLI) were measured. All samples were collected 45 days after heading. The main stem of each plant was cut from the ground. The length of each internode was measured using a straight edge, and the stem diameter was measured at the middle of each internode with a slide caliper. Four randomly chosen plants for each line were measured, and the average for each phenotype was used for the analysis.

### 4.3. QTL Analysis

The 120 CNDH population genetic map was constructed using 788 SSR markers. Among them, 423 SSR markers showed polymorphism, determined using polymorphism analysis. Based on codominant genes in PCR amplification, 222 SSR markers were selected [37]. The total length of the CNDH population genetic map was 2121.7 cM, and the average distance between the markers used to generate the genetic map was 10.6 cM [36]. The Mapmaker version 3.0 was used for evenly distributing markers across 12 of all chromosomes in rice [38]. The naming of QTL is based on the nomenclature proposed by McCough and Doerge [39]. QTL analysis of the length of each internode and stem diameter of each internode was used by the Windows QTL cartographer 2.5 [40]. For whole-genome scanning to detect QTLs, composite interval mapping was employed. The odds score threshold of LOD was set at 2.5 [40] and was used for analysis after entering all of the required data, such as numbers of chromosomes, marker labels, marker genetic distance, data of genotyping, and values of target trait.

### 4.4. Statistical Analysis and Gene Information Analysis

The obtained data were statistically evaluated using GraphPad Prism (version 9.1.1) and the SPSS program (version 26). In particular, statistical analysis was performed by calculating the mean and standard deviation of the measured traits. The frequency distribution graph was analyzed and plotted by GraphPad Prism. Pearson’s correlation was used for correlation analysis by the SPSS program. According to the results of QTL analysis, RiceXpro (https://ricexpro.dna.affrc.go.jp/ (accessed on 5 May 2021) and RAP-DB (https://rapdb.dna.affrc.go.jp/ (accessed on 8 May 2021)) were used to further screen candidate genes and to create a physical map [41]. ORFs were found in SSR markers, and candidate genes were annotated and classified by gene function. For multiple homologous sequences, the NCBI and BioEdit 7.0 comparison was used [42]. The STRING (version 11.0) (https://string-db.org/ (accessed on 8 May 2021)) database was used for the analysis of the protein and protein interaction association network [43].

## 5. Conclusions

Current findings suggest that the cytochrome P450 family gene (*OsCYPq1*) has functional involvement in the stem diameter or internode elongation. The evidence of our current finding supports the idea that (*OsCYPq1*) and its closest paralogs participate in a pathway that controls the stem and internode elongation and ovule integument development. This may also correlate with GA biosynthesis and offers an alternative method for regulating GA-mediated biosynthesis to develop more desirable traits in agriculture. Therefore, the cytochrome P450 gene (*OsCYPq1*) will be most suitable as the target gene to generate mutant semi-dwarf rice and reduce internode elongation, with more productive yield and resistance to lodging.

## Figures and Tables

**Table 1:** Comparative analysis of the length of each internode and the stem diameter of each internode in 120 CNDH population and their parents “Cheongcheong” and “Nagdong”.

Plant Traits	Year	Parents		DH Population
		Cheongcheong	Nagdong.	
LP (cm)	2019	21.9 ± 1.0	18.6 ± 1.1	19.5 ± 2.4
	2020	21.5 ± 0.7	20.6 ± 2.2	19.5 ± 2.5
LUI (cm)	2019	32.5 ± 1.1	30.8 ± 1.3	30.1 ± 5.2
	2020	31.2 ± 0.5	30.8 ± 2.6	30.0 ± 5.3
LSI (cm)	2019	18.6 ± 1.2	16.9 ± 1.4	16.6 ± 4.2
	2020	18.3 ± 0.6	15.9 ± 1.1	16.7 ± 4.3
LTI (cm)	2019	13.7 ± 1.1	11.4 ± 1.2	12.2 ± 3.8
	2020	14.8 ± 1.1	12.1 ± 1.8	12.1 ± 3.7
LFI (cm)	2019	7.8 ± 1.0	4.5 ± 0.2	6.8 ± 3.8
	2020	9.4 ± 0.6	5.3 ± 1.3	6.6 ± 3.7
LLI (cm)	2019	2.8 ± 0.5	2.4 ± 0.3	2.1 ± 2.4
	2020	3.5 ± 0.5	2.1 ± 0.3	2.0 ± 2.4
SDUI (mm)	2019	2.5 ± 0.4	1.4 ± 0.1	2.1 ± 0.45
	2020	2.4 ± 0.2	1.8 ± 0.3	2.1 ± 0.45
SDSI (mm)	2019	4.5 ± 0.3	3.0 ± 0.1	3.6 ± 0.6
	2020	4.3 ± 0.5	3.3 ± 0.3	3.5 ± 0.6
SDTI (mm)	2019	6.0 ± 0.5	4.0 ± 0.2	4.3 ± 0.7
	2020	5.9 ± 0.4	4.4 ± 0.4	4.3 ± 0.7
SDFI (mm)	2019	6.6 ± 0.4	4.8 ± 0.2	4.9 ± 0.7
	2020	6.7 ± 0.2	4.8 ± 0.4	4.9 ± 0.7
SDLI (mm)	2019	6.8 ± 0.3	5.0 ± 0.2	4.9 ± 0.7
	2020	6.8 ± 0.1	5.0 ± 0.6	5.1 ± 0.8

Data are presented as mean ± standard deviation. CNDH, Cheongcheong/Nagdong doubled haploid; LP, length of the panicle; LUI, length of the uppermost internode; LSI, length of the second internode; LTI, length of the third internode; LFI, length of the fourth internode; LLI, length of the lowest internode; SDUI, stem diameter at the uppermost internode; SDSI, stem diameter at the second internode; SDTI, stem diameter at the third internode; SDFI, stem diameter at the fourth internode; SDLI, stem diameter at lowest internode.

**Table 2:** Twenty-three related genes were screened from the target interval RM12285-RM212 on chromosome 1.

Gene ID	Description
*Os01g0782200*	Diacylglycerol kinase, catalytic region domain-containing protein.
*Os01g0783800*	Curculin-like (mannose-binding) lectin domain-containing protein.
*Os01g0784900*	Basic helix-loop-helix dimerization region bHLH domain-containing protein.
*Os01g0785400*	GH3 auxin-responsive promoter family protein.
*Os01g0786900*	WD40-like domain-containing protein.
*Os01g0794400*	Thioredoxin domain 2 containing protein.
*Os01g0797600*	Ethylene-responsive element binding factor3 (OsERF3).
*Os01g0799500*	DNA glycosylase family protein.
*Os01g0802700*	Auxin Efflux Carrier family protein.
*Os01g0803900*	Cytochrome P450 family protein.
*Os01g0823100*	Alpha-expansin OsEXPA2.
*Os01g0833100*	NLI interacting factor domain-containing protein.
*Os01g0840300*	Homeobox domain-containing protein.
*Os01g0851100*	Similar to Axi 1 (Auxin-independent growth promoter)-like protein.
*Os01g0853400*	Similar to Coronatine-insensitive protein 1.
*Os01g0854800*	Similar to Cytochrome P450 86A1.
*Os01g0859500*	Similar to Basic leucine zipper protein (Liguleless2).
*Os01g0867100*	Aminoacyl-tRNA synthetase, class Ib domain-containing protein.
*Os01g0868300*	Similar to DNA polymerase alpha catalytic subunit.
*Os01g0876300*	Galactose oxidase, central domain-containing protein.
*Os01g0881900*	Leucine-rich repeat, cysteine-containing containing protein.
*Os01g0883800*	Gibberellin 20 oxidase 2.
*Os01g0898300*	Armadillo-like helical domain-containing protein.

## Data Availability

Not applicable.

## Supplementary Materials

The following are available online at https://www.mdpi.com/article/10.3390/plants10071369/s1, Table S1: Analysis of the correlation between each internode length and stem diameter in the CNDH population, Table S2: QTL related to each internode length and the stem diameter of each internode of the CNDH population.
