# Supplementary material for: Identification of Candidate Gene for Internode Length in Rice to Enhance Resistance to Lodging Using QTL Analysis
Source: Plants (Basel). 2021 Jul 5;10(7):1369. doi: 10.3390/plants10071369 (PMC8309285; doi:10.3390/plants10071369)
Supplement: Supplementary file 1 [file plants-10-01369-s001.zip › plants-1245834-supplementary.pdf]

Table S1. Analysis of the correlation between each internode length and stem diameter in the CNDH population.

Year	Trait	LP	LUI	LSI	LTI	LFI	LLI	SDUI	SDSI	SDTI	SDFI	SDLI
2019	LP	1.000										
	LUI	0.559**	1.000									
	LSI	0.453**	0.761**	1.000								
	LTI	0.301**	0.635**	0.793**	1.000							
	LFI	0.245**	0.481**	0.521**	0.737**	1.000						
	LLI	0.309**	0.392**	0.465**	0.561**	0.674**	1.000					
	SDUI	0.350**	0.137	0.266**	0.209*	0.067	-0.089	1.000				
	SDSI	0.597**	0.461**	0.465**	0.277**	0.270**	0.297**	0.534**	1.000			
	SDTI	0.597**	0.497**	0.527**	0.355**	0.247**	0.294**	0.500**	0.905**	1.000		
	SDFI	0.606**	0.564**	0.574**	0.441**	0.334**	0.322**	0.516**	0.880**	0.952**	1.000	
	SDLI	0.589**	0.555**	0.615**	0.463**	0.351**	0.341**	0.514**	0.855**	0.902**	0.942**	1.000
2020	LP	1.000										
	LUI	0.557**	1.000									
	LSI	0.434**	0.736**	1.000								
	LTI	0.309**	0.634**	0.783**	1.000							
	LFI	0.218*	0.472**	0.540**	0.748**	1.000						
	LLI	0.296**	0.398**	0.487**	0.548**	0.653**	1.000					
	SDUI	0.333**	0.140	0.241**	0.194*	0.032	-0.053	1.000				
	SDSI	0.584**	0.447**	0.477**	0.300**	0.231*	0.328**	0.527**	1.000			
	SDTI	0.553**	0.490**	0.553**	0.427**	0.251**	0.309**	0.503**	0.902**	1.000		
	SDFI	0.587**	0.571**	0.613**	0.513**	0.369**	0.393**	0.505**	0.858**	0.933**	1.000	
	SDLI	0.573**	0.626**	0.644**	0.587**	0.505**	0.545**	0.410**	0.822**	0.866**	0.949**	1.000

** Correlation is significant at the 0.01 level * Correlation is significant at the 0.01 level. LP, length of the panicle; LUI, length of the uppermost internode; LSI, length of the second internode; LTI, length of the third internode; LFI, length of the fourth internode; LLI, length of the lowest internode; SDUI, stem diameter at the uppermost internode; SDSI, stem diameter at the second internode; SDTI, stem diameter at the third internode; SDFI, stem diameter at the fourth internode; SDLI, stem diameter at lowest internode.

Table S2. QTL related to each internode length and the stem diameter of each internode of the CNDH population

Characteristics	Year	Locus	Chromosome	Interval Markers [a]	LOD	Add. Effect [b]	R^2 [c]	Increasing effect [d]
LP	2019	qLP6	6	RM528-RM3765	3.64	0.87	0.28	Cheongcheong
		qLP9	9	RM3769-RM219	2.66	0.89	0.28	Cheongcheong
	2020	qLP6-1	6	RM528-RM3765	3.26	0.86	0.26	Cheongcheong
LUI	2019	qLUI1	1	RM12285-RM212	9.29	2.40	0.53	Cheongcheong
		qLUI2	2	RM13713-RM213	3.33	1.46	0.55	Cheongcheong
		qLUI5	5	RM5311-RM4691	3.59	1.62	0.56	Cheongcheong
		qLUI6	6	RM528-RM3765	2.80	1.24	0.55	Cheongcheong
	2020	qLUI1-1	1	RM12285-RM212	6.08	2.15	0.49	Cheongcheong
		qLUI2-1	2	RM13594-RM213	3.52	1.61	0.49	Cheongcheong
		qLUI5-1	5	RM5311-RM4691	3.52	1.76	0.51	Cheongcheong
		qLUI6-1	6	RM528-RM3765	3.31	1.72	0.49	Cheongcheong
LSI	2019	qLSI1	1	RM12285-RM212	14.63	2.94	0.54	Cheongcheong
		qLSI5	5	RM5311-RM4691	2.89	1.10	0.55	Cheongcheong
		qLSI7	7	RM20967-RM21972	4.09	1.29	0.56	Cheongcheong
	2020	qLSI1-1	1	RM12285-RM212	14.56	2.93	0.56	Cheongcheong
		qLSI6	6	RM50-RM20196	5.36	1.52	0.54	Cheongcheong
		qLSI6-1	6	RM20632-RM345	2.63	0.90	0.59	Cheongcheong
		qLSI7-1	7	RM418-RM21527	3.15	1.08	0.54	Cheongcheong
LTI	2019	qLTI1	1	RM12285-RM212	15.17	2.26	0.57	Cheongcheong
		qLTI6	6	RM19621-RM20196	5.47	1.32	0.61	Cheongcheong

		qLTI11	11	RM27123-RM27161	3.38	1.13	0.59	Cheongcheong
		qLTI12	12	RM247-RM1261	3.52	1.00	0.59	Cheongcheong
	2020	qLTI1-1	1	RM12285-RM212	13.80	2.36	0.57	Cheongcheong
		qLTI2	2	RM13594-RM3512	3.02	1.06	0.57	Cheongcheong
		qLTI6-1	6	RM50-RM20196	4.45	1.20	0.56	Cheongcheong
		qLTI6-2	6	RM439-RM20318	3.65	1.52	0.65	Cheongcheong
LFI	2019	qLFI1	1	RM12285-RM212	7.35	1.80	0.42	Cheongcheong
		qLFI6	6	RM20355-RM20632	5.00	1.57	0.44	Cheongcheong
	2020	qLFI1-1	1	RM12285-RM212	6.27	1.58	0.44	Cheongcheong
		qLFI6-1	6	RM20355-RM20632	5.19	1.53	0.45	Cheongcheong
LLI	2019	qLLI1	1	RM3482-RM212	2.92	0.71	0.44	Cheongcheong
		qLLI3	3	RM14330-RM218	4.03	0.80	0.43	Cheongcheong
	2020	qLLI1-1	1	RM12285-RM11849	3.65	0.77	0.36	Cheongcheong
		qLLI3-1	3	RM14330-RM218	4.35	0.82	0.37	Cheongcheong
SDUI	2019	qSDUI1	1	RM1287-RM11194	3.98	0.16	0.30	Cheongcheong
		qSDUI11	11	RM3428-RM26771	3.63	0.21	0.29	Cheongcheong
	2020	qSDUI1-1	1	RM1287-RM11194	3.54	0.15	0.29	Cheongcheong
		qSDUI11-1	11	RM287-RM27161	2.56	0.16	0.33	Cheongcheong
		qSDUI12	12	RM12-RM247	3.20	0.28	0.69	Cheongcheong
SDSI	2019	qSDSI1	1	RM1297-RM14323	3.50	0.30	0.44	Cheongcheong
		qSDSI11	11	RM6239-RM26771	3.46	0.24	0.31	Cheongcheong
	2020	qSDSI1-1	1	RM1297-RM14323	2.69	0.23	0.35	Cheongcheong
		qSDSI11-1	11	RM6239-RM26771	2.72	0.21	0.29	Cheongcheong

SDTI	2019	qSDTI1	1	RM1297-RM14323	5.84	0.36	0.44	Cheongcheong
		qSDTI8	8	RM404-RM22861	3.08	0.21	0.33	Cheongcheong
		qSDTI11	11	RM6239-RM26771	3.15	0.26	0.32	Cheongcheong
	2020	qSDTI1-1	1	RM1194-RM5459	2.81	0.24	0.33	Cheongcheong
		qSDTI11-1	11	RM6239-RM27242	5.35	0.39	0.39	Cheongcheong
SDFI	2019	qSDFI1	1	RM1297-RM14323	3.83	0.26	0.36	Cheongcheong
		qSDFI6	6	RM345-RM3765	2.57	0.22	0.34	Cheongcheong
		qSDFI8	8	RM404-RM22861	2.61	0.22	0.33	Cheongcheong
		qSDFI11	11	RM6239-RM26771	3.85	0.30	0.34	Cheongcheong
	2020	qSDFI6-1	6	RM20632-RM3765	2.86	0.23	0.35	Cheongcheong
		qSDFI11-1	11	RM6239-RM26771	4.08	0.32	0.37	Cheongcheong
SDLI	2019	qSDLI1	1	RM1297-RM8111	2.86	0.25	0.36	Cheongcheong
		qSDLI2	2	RM6-RM213	2.69	0.24	0.36	Cheongcheong
		qSDLI6	6	RM528-RM3343	4.51	0.33	0.38	Cheongcheong
	2020	qSDLI1-1	1	RM1297-RM8111	3.55	0.27	0.38	Cheongcheong
		qSDLI2-1	2	RM13594-RM213	3.33	0.27	0.38	Cheongcheong
		qSDLI6-1	6	RM528-RM3343	6.92	0.41	0.37	Cheongcheong

LP, length of the panicle; LUI, length of the uppermost internode; LSI, length of the second internode; LTI, length of the third internode; LFI, length of the fourth internode; LLI, length of the lowest internode; SDUI, stem diameter at the uppermost internode; SDSI, stem diameter at the second internode; SDTI, stem diameter at the third internode; SDFI, stem diameter at the fourth internode; SDLI, stem diameter at lowest internode; [a] Interval Marker are those within the significance threshold on each border of the QTL range. [b] Additive effect. [c] Phenotypic variation explains each QTL. [d] Increase allele is the source of the allele causing an increase in the measured trait.
